# Study protocol for single-center, open-label, randomized controlled trial to clarify the preventive efficacy of electrical stimulation for muscle atrophy after trauma

**DOI:** 10.1186/s13063-018-2872-4

**Published:** 2018-09-14

**Authors:** Makiko Yamamoto, Akio Kimura, Kento Takii, Naruaki Otake, Wataru Matsuda, Tatsuki Uemura, Takunori Sato, Kentaro Kobayashi, Ryo Sasaki, Akiyoshi Hagiwara, Junko Fujitani

**Affiliations:** 1 Department of Emergency Medicine and Critical Care, Center Hospital of the National Center for Global Health and Medicine, 1-21-1 Toyama, Shinjuku-ku, Tokyo, Japan; 2 Department of Physical Medicine and Rehabilitation, Center Hospital of the National Center for Global Health and Medicine, 1-21-1 Toyama, Shinjuku-ku, Tokyo, Japan

**Keywords:** Pelvic fracture, Electrical muscle stimulation, Rehabilitation

## Abstract

**Background:**

Management of trauma involves long-term bed rest even when muscle strength in the lower extremities is preserved. Prolonged bed rest reduces muscle mass and causes muscle atrophy. A recent study reported the efficacy of rehabilitation using electrical muscle stimulation (EMS) for muscle strength maintenance in intensive care unit patients with disturbance of consciousness. However, despite the expected benefits of EMS in maintaining muscle strength, little is known about its efficacy in trauma patients.

**Methods/design:**

A single-center, open-label, randomized controlled trial of 40 patients with pelvic fracture to test the effectiveness of 14 days of EMS. The primary outcome will be change in cross-sectional area of the thigh muscle between pre and post intervention, as measured on computed tomography images. We will analyze the primary endpoint by analysis of covariance (ANCOVA) and analyze the secondary endpoints in an exploratory manner.

**Conclusion:**

If our hypothesis is confirmed, this study will provide evidence that the use of EMS can be effective in preventing muscle atrophy.

**Trial registration:**

UMIN registration number: UMIN000030190. Registered on 1 December 2017.

**Electronic supplementary material:**

The online version of this article (10.1186/s13063-018-2872-4) contains supplementary material, which is available to authorized users.

## Background

Management of trauma, such as cervical spinal cord injury and pelvic fracture, involves long-term bed rest even when muscle strength in the lower extremities is preserved. Prolonged bed rest reportedly reduces muscle mass by 6–40% [1]. After just a few months or years of muscle strength impairment and muscle atrophy, muscle strength may not be completely regained. This contributes strongly to a decline in activities of daily living (ADL) [2]. Physiotherapy including resistance exercises and joint exercises can be started while patients are on bed rest. However, these exercises are not sufficient by themselves to maintain muscle strength; load-bearing exercises are required to maintain muscle strength [3].

A recent study reported the efficacy of rehabilitation using electrical muscle stimulation (EMS) for muscle strength maintenance in intensive care unit patients with disturbance of consciousness [4], clinical illness [5], and spinal cord syndromes [6]. However, despite the expected benefits of EMS in maintaining muscle strength, little is known about its efficacy in trauma patients without lower limb injury who are placed on bed rest immediately after trauma even when muscle strength is unaffected.

In this study, we investigate whether EMS plus conventional physiotherapy is more effective than conventional physiotherapy alone for maintaining muscle strength or retarding its impairment in patients with pelvic fracture who are placed on bed rest.

## Methods/design

### Study setting

This trial is conducted at the Center Hospital of National Center for Global Health and Medicine.

### Objective

To investigate whether addition of EMS to conventional rehabilitation retards loss of muscle mass in trauma patients who require bed rest for at least 1 week despite no initial impairment in muscle strength in the lower extremities.

### Trial design

Single-center, open-label, randomized controlled trial.

### Eligibility criteria

#### Inclusion

1. Age 20–90 years, able to provide informed consent themselves or via a legal representative
2. Trauma inpatient with either unstable pelvic fracture or pelvic fracture at the site where load will be applied. Patients with pelvic fracture included those with an AO classification of 61B2.3 (open book), 61B3.1, 61B3.2, 61C2.1, 61C2.2, and 61C2.3, as well as those who were recommended to rest in bed in supine position for at least 1 week as determined by orthopedic surgeons that we consulted.
3. Requires bed rest for at least 1 week
4. Computed tomography (CT) scans of pelvis and lower extremities taken at the time of hospital admission

#### Exclusion

1. Pacemaker (contraindicated for use with EMS devices)
2. History of neuromuscular disorder (poliomyelitis, myasthenia gravis, or Guillain-Barré syndrome) and/or cerebral infarction (paralysis or contracture)
3. Disturbance of consciousness impairing ability to follow instructions
4. Bed-bound or wheelchair-bound, or with femoral neck fracture and/or intertrochanteric femoral fracture
5. Score of 2 or more for intestinal or pancreatic trauma (based on the Abbreviated Injury Scale (AIS) [7])
6. Fracture or skin injury (except for contusion) at the site for EMS application (thigh, knee joint, or ankle joint)
7. Inability to provide informed consent
8. Participation judged to be inappropriate by the physician in charge

Rationale for criteria 1–2: use of EMS may aggravate preexisting conditions or evaluation may not be conducted accurately.

### Interventions

#### Intervention group

For electrical muscle stimulation, we will use the AUTO Tens PRO Rehabili Unit B-SES (belt electrode skeletal muscle electrical stimulation; Medical Device Certification No.: 224AHBZX00015000) and G-TES (general therapeutic electrical stimulator; Medical Device Certification No.: 228AGBZX00036000) devices (both Homer Ion Co. Ltd., Tokyo, Japan). We will wrap the AUTO Tens PRO belt electrodes around the abdomen, proximal aspect of the legs, and ankles for electrical stimulation of the entire lower extremities. Patients will receive one 20-min EMS session daily, consisting of 5-s stimulations (20 Hz) separated by 2-s rest intervals, for 5 days per week for 2 weeks. The output current will be in the range 2–15 mA, and the current will be increased to the maximum tolerable level within 3 days of starting rehabilitation. A co-investigator who is a physical medicine and rehabilitation physician will supervise the 14-day EMS protocol. In parallel, we will provide conventional physiotherapy on the same day as the EMS session (also for 20 min daily). Conventional physiotherapy will consist of (1) hip and ankle joint mobility exercises and (2) lower limb-strengthening exercises in a supine position.

#### Control group

Patients will receive the conventional physiotherapy protocol alone.

### Criteria for discontinuation

1. Failure to elicit palpable muscle contraction at maximum tolerable current after three daily sessions due to patient discomfort
2. Any injury to the skin and/or striated muscle
3. Voluntary withdrawal of consent from the trial

Rationale for criteria 1 and 2: to eliminate the influence of these factors on the evaluation of efficacy and safety.

Rationale for criterion 3: to respect participants’ voluntary decision.

### Outcomes

#### Primary endpoint

- Change in cross-sectional area of thigh muscle between pre and post intervention, as measured on CT images

#### Secondary endpoints

We will measure all secondary endpoints on the same dates that CT imaging is obtained.

- Change in cross-sectional area of calf muscle between pre and post intervention, as measured on CT images
- Reduction in thigh and calf circumference, measured at the same sites as CT image measurements
- Thigh and calf circumference measured on CT images
- Changes in manual muscle testing results

We will obtain baseline CT data of the region including the pelvis and lower extremities at the time of admission (pre intervention, from day − 4 to day 0) and measure cross-sectional area of the thigh and calf muscles. Day 1 is defined as the first day of rehabilitation intervention.

To evaluate the outcomes of intervention, we will obtain post-intervention plain CT data of the region including the pelvis and lower extremities and measure cross-sectional area of the thigh and calf muscles on day 14 of intervention (or before the end of day 17). Specifically, we will obtain the following measurements.

1. Thigh: height at the center of a line linking the greater trochanter and knee joint cleft
  Calf: height at the center of a line linking the fibular head and external condyle
2. Thigh and calf muscle cross-sectional area: we will extract areas with intensity in the range of 30 to 100 Hounsfield Units (HU) from CT slices and measure cross-sectional area to determine change between pre- and post-intervention values

We will obtain CT scans in the supine position with both legs in the neutral position, with a pillow placed under both legs to avoid compression of the posterior aspect of the legs from the CT bed. We will ask patients to relax to minimize the influence of morphological changes due to muscle contraction. The following CT machines will be used:

- Aquilion™ CX TSX-101A/NA, Application Ver. V4.62JR019 (Toshiba Medical Systems Corp., Tokyo, Japan)
- SOMATOM Definition Flash VA44A, Somaris/7 Syngo CT 2012B
  WinNT 6.1, Service Pack 1, VA44A_08_P16 (Siemens Healthcare, Forchheim, Germany)
- Aquilion ONE™ TSX-301A/2A, Application Ver. V4.7JR004 (Toshiba Medical Systems Corp.)
- Discovery CT 750HD, Application Software, 11 MW44.11.V40_PS_HD64_G_GTL (General Electric Healthcare, Waukesha, WI, USA)

### Sample size

Target sample size: 50 patients (25 each in the intervention group and control group).

### Rationale

In a previous study of disuse muscle atrophy in patients with consciousness disturbance in the intensive care unit (ICU) [4], change in cross-sectional area was found to be 0.85 on average after 1 week of conventional rehabilitation (without EMS). The ratio of cross-sectional area pre and post intervention changed by 0.85 after 2 weeks of EMS-integrated rehabilitation (EMS group) compared with 0.72 after 2 weeks of conventional rehabilitation (control group), for a difference of 0.13 between the groups. For the present study, we recalculated the sample size for analysis of covariance (ANCOVA) with the standard deviation of 0.15 for both groups, alpha error of 0.05, power of 0.8, and covariance of 1. A sample size of 44 was required based on the calculation. Thus, we revised the manuscript to include 25 patients for each group.

To calculate sample size for the present study, we determined that approximately 25 pelvic fracture patients required 2-week hospitalization at the Department of Emergency Medicine and Critical Care, Center Hospital of the National Center for Global and Medicine between 2015 and 2016, with no dropouts including those due to death. Thus, a total of 50 patients (25 in each group) is required for the present study.

## Methods

### Assignment of intervention

#### Enrollment procedure

Patients who are deemed eligible for the study according to the inclusion and exclusion criteria will be enrolled in the study within 1–2 days after hospital admission. Before entry, patients themselves must sign informed consent forms. In the case that patients can follow instructions, but dementia or mental retardation makes the voluntary nature of their actions unclear, their legal representatives (third-degree relatives and legal guardians) must provide written informed consent.

### Allocation

A computer-generated random number table will be prepared by an individual who is not an investigator. The table will be stored in a designated safe by the coordinator of the outpatient division of the Department of Emergency Medicine and Critical Care, who is not involved in the study. Study investigators who are the physician in charge of admitted patients satisfying the enrollment criteria will report the admissions to the coordinator. The coordinator will then allocate patients to either of the groups using the prepared random number table and will notify the investigators of the allocation results.

### Participant data collection

**Fig. 1:**
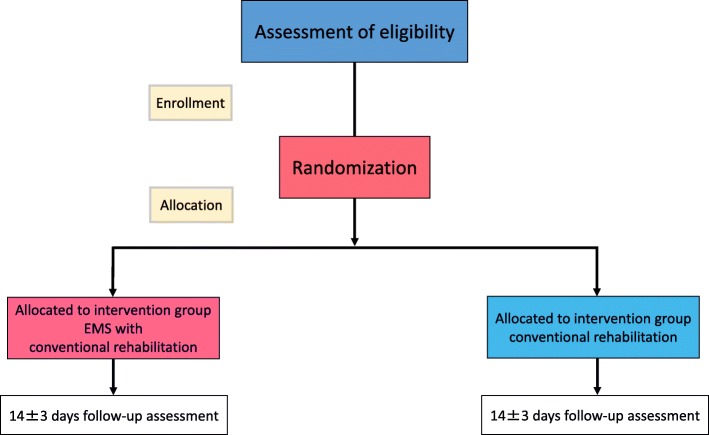
Study flowchart

**Fig. 2:**
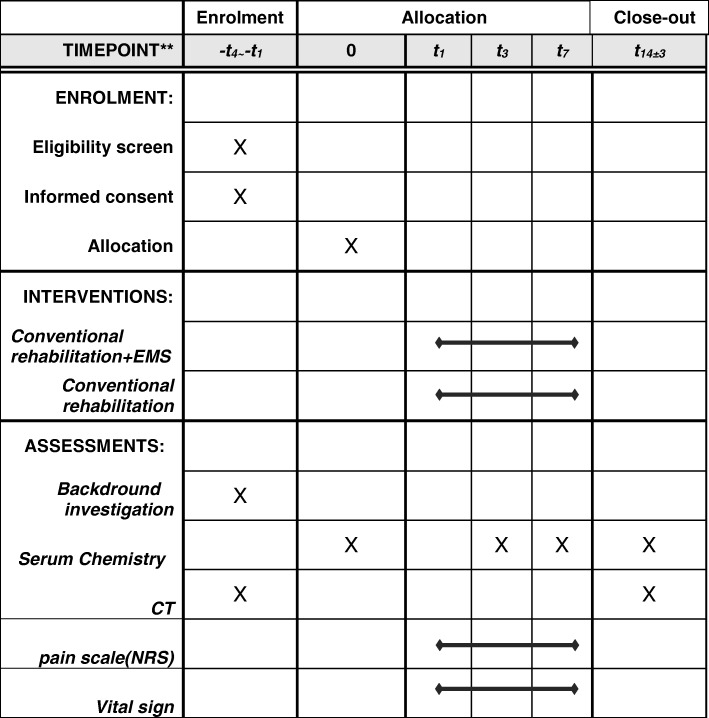
Treatment schedule and outcome measures

See Fig. 1 for details of the data collection schedule. Figure 2 shows the Standard Protocol Items: Recommendations for Interventional Trials (SPIRIT) schedule for the trial protocol.

1. Participant background: age, sex, medical history, height, weight, diagnosis at time of admission (type of trauma, AIS score, ADL, Functional Independence Measure score [8], and Barthel ADL Index [9])
2. Physical findings for the EMS group only: blood pressure, heart rate, oxygen saturation, and level of consciousness before starting EMS, 10 min after starting EMS, and on completion of EMS; pain level using a numerical rating scale [10] during EMS; thigh and calf circumference at the time of admission (pre intervention) and on days 14–17 (post intervention); manual muscle testing
3. Imaging diagnosis: measurements of cross-sectional thigh and calf area on plain CT images taken at the time of admission (from day −  4 to day 0) and at the end of rehabilitation (days 14–17)
4. Clinical testing: creatinine kinase (CK) level on days 0, 3, 7, and 14–17
5. Functional Independence Measure score and Barthel ADL Index at 1 and 2 months after the injury

### Statistical analysis

For all participants who satisfy the inclusion criteria and start receiving the test intervention, as a definitive statistical analysis model, ANCOVA (regression model) was used to evaluate the effect of the treatment. The outcome measure was the rate of reduction, and independent variables included the treatment method, possible confounders in randomization, and baseline measure of muscle mass and analyze the secondary endpoints in an exploratory manner.

### Missing data

While missing data were not dealt with in the present study, data may be complemented after some process as appropriate if there are a certain number of missing values.

### Blinding

Assessor, participants, treatment team, and statisticians were unblinded.

### Post-trial care

Conventional therapy will be continued in consultation with the physicians in charge.

### Data management

#### Monitoring

A monitoring officer will conduct monitoring before, during, and after completion, termination, or withdrawal from the study.

### Harms and benefits

1. Potential burdens and risks

EMS is widely used in patients with cardiac disease or diabetes mellitus, and its sufficient safety has been proven. However, the adverse side effects listed below have been reported, and thus will be monitored for continuously during the study. If they do occur, the physician in charge will provide appropriate care and treatment.

#### Potential side effects

- Skin injury and rash at the site of belt attachment
- Aggravation of pain at the site of fracture
- Pain during EMS

2. Expected benefits

The benefits of conventional physiotherapy are already well established in rehabilitation. Also, EMS is reported to prevent muscle atrophy. Thus, addition of EMS to the conventional physiotherapy with proven benefits may enhance the therapeutic effect.

### Evaluation and reporting of adverse events

#### Definition of adverse events

1. Serious adverse events
  We will record any adverse event listed below, occurring in the period between the date of providing informed consent and day 14, irrespective of the presence or absence of a causal relationship. Also, we will promptly report any occurrence to the head of the research institute via the reporting division of the hospital within 7 days after the serious adverse event is first noticed.
  Serious adverse events:
  1. Resulting in death
  2. Being life-threatening
  3. Requiring inpatient hospitalization or prolongation of existing hospitalization
  4. Resulting in persistent or significant disability/incapacity
  5. Resulting in congenital anomaly
2. Other adverse events
  The therapeutic devices that will be used in this study have already been approved and are routinely used in the clinical setting. Thus, we will not record non-serious adverse events.
  We will use the designated case report form to record case information. We will register the case information digitally, with the principal investigator assigning the case number. After registration, the principal investigator and an analyst will analyze individual cases. They will pass the analysis report to the planning and strategy manager.

### Confidentiality

1. Access to the personal information and data collected in this study will be strictly limited to investigators of this study. The data collected will be for the purpose of conducting the study only. Investigators will handle personal information carefully. The principal investigator will put in place measures for appropriate information and data handling
2. Physicians in charge will initially collect data on paper. They will then register the collected data in the study database. The study database will not contain personally identifiable information
3. The study database will be stored on the hard disk drive of a password-protected computer kept in a lockable room. The study group will have a key to the room and maintain security of the computer password. The study group will appropriately handle research-related printed documents, memos, and other details, for example, by viewing them in an isolated room to avoid exposure to a third party and storing them in a lockable cabinet. The study group will minimize the use of portable electronic media, but if use is absolutely necessary, members must notify the principal investigator in advance of use and handle the media with extreme caution
4. The principal investigator is responsible for storage of the anonymized random number table and for supervising the format. Once data are fixed in the study database, the principal investigator will hand the random number table to the center’s planning and strategy manager and no copies will be held by the study group
5. Personal information and data collected during the study will be kept for 5 years from the time of publication of the main study outcomes before disposal. Both printed and electronic materials will be destroyed so that the data are unreadable before disposal. Some writable media may be reused after overwriting the original data with dummy data to make the original data irretrievable
6. When disclosing study outcomes, study members will take all due care to prevent re-identification of de-identified data

### Protocol amendments

The study will be conducted after the study plan defining the study protocol is assessed and approved by the Ethics Committees of all participating centers. The Ethics Committees will review the study plan when protocol amendments are deemed necessary and must approve all protocol amendments.

### Dissemination policy

Study outcomes will be reported at professional meetings and submitted to a scientific journal for publication.

## Discussion

Population aging is progressing worldwide, and the number of aged patients with fracture of the femur or pelvis due to falls or traffic accidents has been increasing. Muscle atrophy in patients with femoral fracture can be minimized by physiotherapy with a specific load early after operative fixation if performed soon after the accident. However, regardless of early fixation, patients with severe pelvic fracture may need long-term bed rest because a gravitational load cannot be applied for a relatively long time. This causes marked reduction of muscle mass of the lower extremities. EMS is applicable to such patients, and our study may provide evidence that EMS can prevent muscle atrophy to some extent and will provide an alternative to rehabilitation for these patients. Therefore, it is worthwhile to conduct this study.

Finally, we discuss the potential limitations of the study. First, we think that differences among individuals may emerge when muscles contractions cause pain. To solve this problem, we will provide patients with sufficient analgesics, gradually increasing the dose as needed. A second limitation is that this study is open-labeled. We think that there is a possibility that patients of controls have slower rehabilitation than those of receiving EMS, and thus bias due to unblinding might persist. However, it is impossible to hide which allocated patients receive EMS because patients can feel when their muscles are stimulated (Additional file 1).

## Trial status

Protocol version: 1.1

Study period: 14 November 2017 to 31 December 2019.

## Additional file

Additional file 1: Study protocol for single-center, open-label, randomized controlled trial to clarify the preventive efficacy of electrical stimulation for muscle atrophy after trauma. (DOCX 53 kb)

## Abbreviations

ADL: Activity of daily living
AIS: Abbreviated Injury Scale
ANCOVA: Analysis of covariance
CK: Creatine kinase
CT: Computed tomography
EMS: Electrical muscle stimulation
HU: Hounsfield Unit
UMIN: University Hospital Medical Information Network
